# 

**Published:** 1859-02

**Authors:** 


### RECEIPTS FOR JOURNAL UP TO MARCH 1st, 1859.

### Illinois.

| | | |
|---|---|---|
| Dr. A. Berchleman, | vol. 2, 1859, | $2 00 |
| " E. T. Nichols, | " " | 2 00 |
| " L. A. Engle, | " " | 2 00 |
| " J. Blount, | " " | 2 00 |
| " Jas. O. Roman, | " " | 2 00 |
| " J. Woodworth, | " " | 2 00 |
| " E. P. Cook, | " " | 2 00 |
| " W. Wynn, | " " | 2 00 |
| " C. D. Henton, | " " | 2 00 |
| " T. S. Black, | " " | 2 00 |
| " O. Q. Herrick, (ex. cop.) | " " | 4 00 |
| " J. B. Meigs, | " " | 2 00 |
| " J. W. Trabue, | " " | 2 00 |
| " Levi Propst, | " " | 2 00 |
| " Chas. F. Leitchenberger, | vols. 1 & 2, 1858 & 1859, | 3 00 |
| " Thos. B. Trover, | vol. 1, 1858, | 2 00 |
| " A. J. Randal, | " " | 2 00 |

### Wisconsin.

| | | |
|---|---|---|
| Dr. Lafayette Lake, | vol. 2, 1859, | $2 00 |
| " P. R. Slingsby, | " " | 2 00 |
| " N. L. Cook, | vol. 6, 1857, | 2 00 |
| " L. G. Armstrong, | vol. 2, 1859, | 2 00 |

### Indiana.

| | | |
|---|---|---|
| Dr. R. Q. Wilson, | vol. 2, 1859, | $2 00 |
| " J. H. Harriman, | " " | 2 00 |
| " C. M. Richmond, | vol. 6, 1857, | 1 00 |
| " Miller & Owen, | vol. 2, 1859, | 2 00 |
| " Robt. Winton, | " " | 2 00 |
| " W. C. Willard, | " " | 2 00 |
| " J. S. Collings, | vol. 1, 1858, | 2 00 |

### Michigan.

| | | |
|---|---|---|
| Dr. J. M. Phillips, | vol. 2, 1859, | $2 00 |
| " Edwin Stewart, | " " | 2 00 |
| " C. S. Tucker, | " " | 2 00 |

### Iowa.

| | | |
|---|---|---|
| Dr. R. W. Hall, | vols. 1&2, 1858&9, | $4 00 |
| " E. Clifford, | vol. 2, 1859, | 2 00 |
| " John Low, | " " | 2 00 |
| " J. N. B. Elliott, | vol. 1, 1858, | 2 00 |
| " Hamilton Love, | vol. 2, 1859, | 2 00 |
| " R. W Gavren, | vols. 6, 6, & 1, 1856, 1857 & 1858, | 6 00 |

### Kentucky.

| | | |
|---|---|---|
| Dr. Benj. F. Estes, | vol. 2, 1859, | $2 00 |

### Missouri.

| | | |
|---|---|---|
| Dr. R. B. Ray, | vol. 1, 1858, | $2 00 |

